# Supplementary material for: Occurrence and Source Identification of Polychlorinated Dibenzo-p-dioxins and Dibenzofurans and Polychlorinated Biphenyls in Surface Sediments from Liangshui River in Beijing, China
Source: Int J Environ Res Public Health. 2022 Dec 8;19(24):16465. doi: 10.3390/ijerph192416465 (PMC9779105; doi:10.3390/ijerph192416465)
Supplement: Supplementary file 1 [file ijerph-19-16465-s001.zip › ijerph-1996909-supplementary.pdf]

International Journal of
Environmental Research and Public Health

MDPI

Supplementary Materials

Occurrence and Source Identification of Polychlorinated Dibenzo-p-dioxins and Dibenzofurans and Polychlorinated Biphenyls in Surface Sediments from Liangshui River in Beijing, China

Honghua Li [1], Pu Wang [2], Yongming Ju [3,4], Wenjuan Li [2], Ruiqiang Yang [1], Gang Li [1], Wenqiang Ren [1], Jie Li [1] and Qinghua Zhang [1,5,*]

1 State Key Laboratory of Environmental Chemistry and Ecotoxicology, Research Center for Eco-Environmental Sciences, Chinese Academy of Sciences, Beijing 100085, China
2 Hubei Key Laboratory of Industrial Fume and Dust Pollution Control, School of Environment and Health, Jianghan University, Wuhan 430056, China
3 Laboratory of Pesticide Environmental Assessment and Pollution Control, Nanjing Institute of Environmental Sciences, Ministry of Ecology and Environment (MEE), Nanjing 210042, China
4 The Key Laboratory of Water and Air Pollution Control of Guangdong Province, South China Institute of Environmental Sciences, Ministry of Ecology and Environment (MEE), Guangzhou 510655, China
5 University of Chinese Academy of Sciences, Beijing 100049, China
* Correspondence: qhzhang@rcees.ac.cn; Tel.: +86-10-6284-9818

Table S1. Comparison of PCDD/Fs and PCBs in sediments from different regions.

Locations	Year	n	PCDD/Fs (pg g⁻¹ dw)	PCBs (pg g⁻¹ dw)	PCDD/Fs (pg TEQ g⁻¹ dw)	PCBs (pg TEQ g⁻¹ dw)	Reference
Taihu Lake, China	2002	10	134.4–284.9 (206.6)	2707.2–5964.1 (3723.0)	0.84–3.71 [b] (2.18, sum of PCDD/Fs and PCBs)	–	[41] [f]
Haihe River, China	2003	10	151–11,546 (2013)	775–7473 (3077)	1.8–20 [c] (7.7)	0.1–0.5 [c] (0.27)	[17]
Dagu Drainage River, China	2003	3	1962–556,961 (201290)	44266–153,727 (89,439)	19–975 [c] (424)	1.1–21 [c] (8.3)	[17]
Daliao River Estuary, China	2007	35	11.3–133.2 (61.2)	1971–37,632 (5917)	0.3–3.08 [c] (1.3)	0.04–1.7 [c] (0.34)	[34]
Liaohe River, China	2004	15	13.7–458.5 (121.9)	1853–1,075,606 (232,868)	0.24–27.49 [c] (3.01)	0.015–0.99 [c] (0.33)	[20]
Liaohe River, China	2003–2004	2	9–10 (10)	290–630 (460)	0.002 [b]	0.001 [b]	[21]
Yellow River, China	2003–2004	7	10–180 (53)	960–12,800 (3579)	0.002–1.89 [b] (0.29)	0.001–0.08 [b] (0.027)	[21]
Tarim River, China	2003–2004	3	10–40 (27)	1940–2960 (2283)	0.001–0.1 [b] (0.060)	0.002–0.04 [b] (0.021)	[21]
Ertix River, China	2003–2004	1	20	1190	0.003 [b]	0.02 [b]	[21]
Yangtze River, China	2003–2004	9	30–330 (159)	540–7710 (3180)	0.004–0.58 [b] (0.043)	0.001–0.16 [b] (0.04)	[21]
Haihe River, China	2003–2004	3	10–90 (47)	2820–5360 (3840)	0.002–0.23 [b] (0.086)	0.03–0.1 [b] (0.053)	[21]
Pearl River, China	2003–2004	12	230–6490 (3192)	2830–21,700 (7393)	0.05–4.18 [b] (1.32)	0.02–0.47 [b] (0.15)	[21]
Pearl River Delta, China	2011	14	–	16,150–477,850	–	0.12–639.52	[27]
East River, China	2007	9	2100–5400 (3600)	48–270 (131)	2.4–9.1 [b] (4.7)	0.039–0.44 (0.18)	[33]
Jiaojiang Estuary, China	2008	23	–	4930–108,790 (29,080)	–	–	[55]
Dianchi Lake, China	2008	10	–	600–2400 (1200)	–	–	[40]
Pound in China	2009	1	33,718	–	128 [b]	–	[56]
Reservoir in Northern Taiwan [d]	2000	1	–	–	0.663–4.61 [b] (2.8)	0.041–0.261 [b] (0.11)	[57]
Coastal Seas and Lakes in Shandong, China	–	5	47.7–147 (105)	21.3–54.4 (36)	0.11–0.80 [b] (0.52)	0.03–0.08 [b] (0.054)	[38]
Han River, Korea	2005–2006	18	23.1–368 (131)	41.5–4530 (548)	0.679–10.5 [b] (3.39)	0.00514–0.626 [b] (0.19)	[36]
Hyeongsan River, Korea	2001	6	4.8–1600 (480)	<1000–170,000 (62,000)	0.38–1037 [b]	0.05–3.7 [b]	[58]
Lake Shihwa, Korea	2008	34	40–100,000	50–335,000	0.1–1600 [c]	0.004–185 [c]	[25]
Coastal Lagoons, Vietnam	2002–2006	13	197–2919 (1012)	–	0.25–5.24 [c] (2.0)	–	[59]
Black Sea, Turkey	–	16	3.47–174.74	700–23,720	–	–	[23]
River Elbe, Germany [d]	1995	2	16,054–32,624	329–1785	173–433 [b]	1.7–13 [b]	[18]
Mondego Estuary, Portugal	2009	1	110	199	0.82 [c]	0.16 [c]	[37]
Pounds in France	2008	10	–	300–3500 (2400)	–	–	[29]
Southern North Sea and English Channel	2001	5	0.124–3.156	0.102	–	–	[26]
Grenlandsfjords, Norwegian	2000	9	25,000–733,000 (210,000)	–	310–10,000 [a] (3300)	–	[19]
Great Lakes, North American	2010–2014	40	150–3540	1400–64,300	1.1–98 [c]	–	[22]
Houston Ship Channel, USA	2002–2003	98	–	4180–4,601,000 (168,000)	–	–	[28]
Bering Sea	2008	7	–	22–150 (71)	–	–	[31]

Guaratuba Bay, Brazil	2010	17	–	<LQ [e]–5620	–	–	[30]
Lake Victoria, East Africa	2011	25	3.36–51.4	21.4–247	0.08–4.24 [c]	0.02–0.21 [c]	[24]
River in South Africa	2006	5	–	–	0.93–13.44 [c] (4.4)	0.08–0.58 [c] (0.26)	[35]
Liangshui River, China	2013	27	3.5–3019 (184)	319–5949 (1958)	0.0011–5.1 [c] (0.99)	0.0074–1.4 [c] (0.48)	This study

Note: [a], I-TEQ (1989); [b], WHO-TEQ (1998); [c], WHO-TEQ (2005); [d], Sediment core; [e], Below the limit of quantification; [f], Reference numbers in the manuscript; –, Not available; Mean value in parentheses.

Table S2. Compositions of PCDD/F congeners in possible sources.

Possible sources	2,3,7,8-TCDF	1,2,3,7,8-PeCDF	2,3,4,7,8-PeCDF	1,2,3,4,7,8-HxCDF	1,2,3,6,7,8-HxCDF	2,3,4,6,7,8-HxCDF	1,2,3,7,8,9-HxCDF	1,2,3,4,6,7,8-HpCDF	1,2,3,4,7,8,9-HpCDF
PCP (ng g^{-1})	3.0	12.9	3.8	97.7	13.7	0.1	1.3	230	29.6
PCP-Na (ng g^{-1})	3.1	40.9	2.4	76.1	9.4	0.32	0.93	135	18.3
STEEL (fg m^{-3})	62.04	63.22	88.70	82.65	66.57	67.61	4.35	234.57	31.74
Al (ng Nm^{-3})	0.18	0.04	0.35	0.1	0.09	0.13	0.03	0.1	0.02
Cu (ng Nm^{-3})	0.42	0.11	3.18	0.97	0.99	1.76	0.48	1.42	0.28
IOS (ng Nm^{-3})	0.5	0.65	1.05	0.7	0.65	0.8	0.25	1.05	0.2
MSWI-FS (ng kg^{-1} dw)	0.5	0.75	0.74	1.35	0.9	0.86	0.26	4.06	0.33
MSWI-PS (ng kg^{-1} dw)	0.26	0.3	0.23	0.47	0.41	0.37	0.05	1.52	0.19
MSWI-F (ng Nm^{-3})	0.006	0.007	0.017	0.008	0.008	0.009	0.002	0.017	0.001
MSWI-FA (ng kg^{-1} dw)	223	168	222	100	113	105	32.4	179	25.5
WWPM (pg l^{-1})	121.5	65.3	26.8	0	8.4	0	0	0	0
HCB (ng kg^{-1} fuel)	9.3	2.6	1.8	0.7	0.6	0	0.5	1.3	0.3
ASOB (pg Nm^{-3})	0.117	0.172	0.278	0.274	0.257	0.0427	0.317	0.986	0.123

Possible sources	OCDF	2,3,7,8-TCDD	1,2,3,7,8-PeCDD	1,2,3,4,7,8-HxCDD	1,2,3,6,7,8-HxCDD	1,2,3,7,8,9-HxCDD	1,2,3,4,6,7,8-HpCDD	OCDD	Reference
PCP (ng g^{-1})	3674	14.1	2.2	351	0	3.5	2473	22,026	[44] [a]
PCP-Na (ng g^{-1})	1647	4	2.1	244	13.8	2.1	1702	12,514	[44]
STEEL (fg m^{-3})	194.70	0.87	1.91	3.70	10.57	6.57	99.39	332.83	[45]
Al (ng Nm^{-3})	0.08	0.05	0.03	0.01	0.01	0.01	0.01	0.01	[20]
Cu (ng Nm^{-3})	0.57	0.49	0.45	0.14	0.19	0.14	0.22	0.11	[20]
IOS (ng Nm^{-3})	0.25	0.05	0.1	0.12	0.2	0.12	0.45	0.35	[20]
MSWI-FS (ng kg^{-1} dw)	2.83	0.07	0.28	0.24	0.51	0.57	4.99	36	[46]
MSWI-PS (ng kg^{-1} dw)	1.22	0.06	0.12	0.16	0.32	0.4	3.68	58.1	[46]
MSWI-F (ng Nm^{-3})	0.016	0.001	0.003	0.002	0.004	0.003	0.017	0.052	[46]
MSWI-FA (ng kg^{-1} dw)	30.5	23.6	42	20.1	24.4	24.5	95.1	104	[46]

WWPM (pg l^{-1})	19.6	229.5	100.5	24	16.5	15	54.4	63	[47]
HCB (ng kg^{-1} fuel)	1.4	0.5	0.4	0.2	0.3	0	4.1	33	[45]
ASOB (pg Nm^{-3})	0.569	0.0128	0.0523	0.0461	0.0964	0.0836	0.527	0.684	[49]

Note: [a], Reference numbers in the manuscript.

Table S3. Compositions of dl-PCB congeners in possible sources.

**Possible sources**	**PCB-77**	**PCB-81**	**PCB-126**	**PCB-169**	**PCB-105**	**PCB-114**	**PCB-118**	**PCB-123**	**PCB-156**	**PCB-157**	**PCB-167**	**PCB-189**	**Reference**
HCB (ng kg^{-1} fuel)	21	–	1.8	0.2	40	2.3	96	6.6	13	2.5	5.2	0.9	[48] [a]
HWB (ng kg^{-1} fuel)	2.7	–	0.2	0.1	5.3	0.3	8.5	1.2	0.9	0.2	0.4	nd	[48]
KC300 (%)	0.19	0.0086	0.001	nd	0.14	0.014	0.25	0.0095	0.006	0.0015	0.0025	0.001	[51] [b]
KC400 (%)	0.28	0.011	0.0047	<0.001	1.1	0.085	1.8	0.051	0.039	0.0096	0.016	<0.001	[51]
KC600 (%)	0.039	0.0018	0.0019	<0.001	1.9	0.1	5.8	0.14	0.67	0.14	0.3	0.024	[51]
Ar1221 (%)	0.011	nd	nd	nd	0.01	nd	0.015	nd	nd	nd	nd	nd	[52]
Ar1232 (%)	0.16	nd	nd	nd	0.23	0.023	0.38	0.025	0.027	0.0048	0.0081	nd	[52]
Ar1242 (%)	0.31	nd	0.0084	nd	0.41	0.057	0.59	0.029	0.019	0.0049	0.0076	nd	[52]
Ar1248 (%)	0.54	nd	0.01	nd	0.99	0.11	1.7	0.06	0.058	0.0088	0.013	0.013	[52]
Ar1254 (%)	0.098	nd	nd	nd	2.6	0.16	6.9	0.26	0.91	0.18	0.32	0.032	[52]
Al (ng Nm^{-3})	0.0017	0.0038	0.1879	0.0197	0.0016	0.0002	0.0038	0.0006	0.0003	0.0001	0.0002	0.0001	[20]
Cu (ng Nm^{-3})	0.0004	0.0007	0.2273	0.0266	0.0002	0.0001	0.0007	0.0001	0.0001	0	0	0.0001	[20]
IOS (ng Nm^{-3})	2	0.35	0.7	0.1	2.5	0.3	6.6	0.4	0.6	0.25	0.35	0.2	[20]
WWTP (pg g^{-1} dw)	137.025	9.7125	5.1	1.3	332.125	31.7625	693	65.025	75.275	18.0375	27.5875	6.4	[39]
STEEL (fg m^{-3})	1274	113	49	12	749	81	1522	158	102	29	29	22	[45]
MSW (ng g^{-1} wet)	0.043	0.003	0.0038	0.0008	0.12	0.027	0.29	0.0041	0.041	0.01	0.015	0.0048	[53]

Note: [a], Reference numbers in the manuscript.

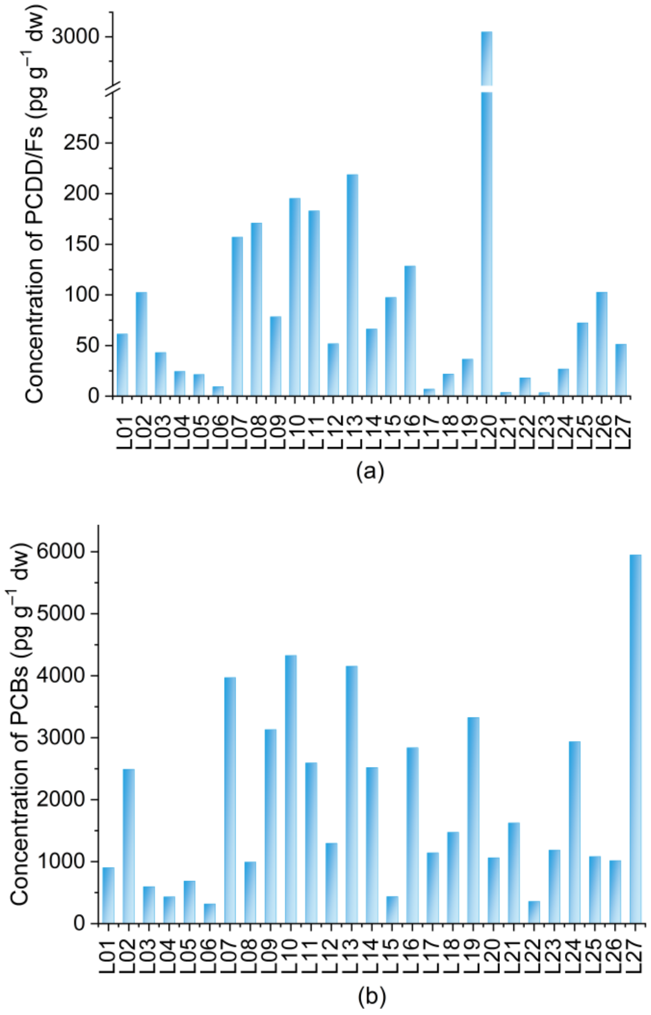

Figure S1. Distribution of PCDD/F (a) and PCB (b) concentrations in the sediments from LSR.
